# Measuring Surgery Outcomes of Lung Cancer Patients with Concomitant Pulmonary Fibrosis: A Review of the Literature

**DOI:** 10.3390/cancers10070223

**Published:** 2018-07-04

**Authors:** Taichiro Goto

**Affiliations:** Lung Cancer and Respiratory Disease Center, Yamanashi Central Hospital, Kofu 400-8506, Japan; taichiro@1997.jukuin.keio.ac.jp; Tel.: +81-55-253-7111

**Keywords:** lung cancer, idiopathic pulmonary fibrosis, surgery, acute exacerbation, interstitial lung diseases

## Abstract

Idiopathic pulmonary fibrosis (IPF), the most common form of idiopathic interstitial pneumonias, often progresses to restrictive respiratory disturbance and mortality, typically within 10 years. IPF frequently coexists with lung cancer, and the combination of these two disease entities is far more difficult to treat than either lung cancer or IPF alone. In particular, surgery for lung cancer with IPF in the background increases postoperative morbidity and mortality by exacerbating pre-existing IPF, i.e., acute exacerbation of IPF (AEIPF). Furthermore, the long-term outcome after lung cancer surgery is considerably worsened by the presence of IPF. We present here a comprehensive review of AEIPF and the long-term outcomes after surgery.

## 1. Introduction

Interstitial pneumonia (IP) is a known risk factor for lung cancer. The prevalence of IP in patients with lung cancer is approximately 4–8% [1,2,3]. Idiopathic pulmonary fibrosis (IPF) is considered an independent risk factor for lung cancer [4,5,6,7], such that patients with IPF experience a 7- to 14-fold greater risk of lung cancer than the general population [8]. Some reports have shown that approximately 10% of the deaths among IPF patients are due to lung cancer [4]. Idiopathic interstitial pneumonia (IIP) constitutes the most common form of interstitial lung disease associated with lung cancer, but this can be a challenging differential diagnosis, as IIP is often overlooked in the diagnosis and treatment of lung cancer. Thus, IP evaluation in clinical cases of IIP-associated lung cancer comprises a heterogenous disease group consisting of various pathological conditions with different risks for acute exacerbation (AE) spread over multiple disease stages; thus, the interpretation of study results is extremely complicated. Anti-cancer therapies for patients with IP-associated lung cancer include radiotherapy, chemotherapy and surgical therapy, any of which can cause AE and/or fatal complications. Thus, careful selection of a suitable treatment strategy is required. The aims of this article are to review the current status regarding the outcomes of lung resection for lung cancer patients with concomitant IPF and to address the prediction and prevention of AE, focusing on the outlook for the future. The available literature was examined using references from MEDLINE. The search strategy included the following keywords: ‘lung cancer’, ‘idiopathic pulmonary fibrosis’, ‘interstitial lung diseases’, ‘surgery’, ‘acute exerbation’, ‘complication’, ‘morbidity’, ‘mortality’, ‘recurrence’, ‘prognosis’ and ‘outcome’, and included articles published from August 1996 to May 2018. A total of 63 papers were ultimately accepted for this review.

## 2. Interstitial Pneumonia-Associated Lung Cancer and Surgery

IPF is the most common form of IIP and is associated with poor prognosis, with a mean post-diagnosis survival of 3–5 years [9,10,11]. IPF frequently coexists with lung cancer [4,5,6]. In IPF patients and IIP patients with lung cancer, p53 overexpression/mutation is frequently detected in fibrotic foci and the surrounding alveolar and bronchiolar epithelia, along with mutations of *K-ras* and the tumor suppressor gene *FHIT* [12,13,14,15,16]. These data suggest that IPF itself is a precancerous lesion. IPF-associated lung cancer is often encountered in daily clinical practice and is associated with difficulty in treatment selection due to poor prognosis of IIP, particularly IPF [7]. Furthermore, chemotherapy, radiotherapy and surgical therapy can all trigger AE [7]. Compared with lung cancer patients without IP, patients with IP-associated lung cancer are more likely to be older male and heavy smokers, with a histological diagnosis of squamous cell carcinoma [17,18]. This carcinoma commonly occurs in the distal dorsal side of the lower lobe, which tends to be severely affected by IPF, suggesting that IPF-associated persistent chronic inflammation plays a role in cancer development. Acute exacerbation of idiopathic pulmonary fibrosis (AEIPF) is characterized by diffuse and rapid alveolar damage on a background of IPF, which most likely occurs as a result of a massive lung injury from an unknown etiologic agent. Acute respiratory distress syndrome (ARDS) can also occur after surgery for lung cancer, with especially high incidence and mortality rates after pneumonectomy [19,20,21]. Thus, postoperative AE of IP can be a condition in which ARDS has occurred in a patient with IP-associated lung cancer who has undergone surgery. A Japanese nationwide survey for respiratory surgeries conducted in 2009 showed that 268 surgery-related deaths occurred after surgery in 231,301 cases of lung cancer (0.86%) [22]. The most common cause of death was AE of IP in 68 cases, constituting 25% of all surgery-related deaths. Thus, postoperative AE of IP remains an important problem that needs to be addressed.

## 3. Pathology of the Microscopic Usual Interstitial Pneumonia Pattern

**Figure 1:**
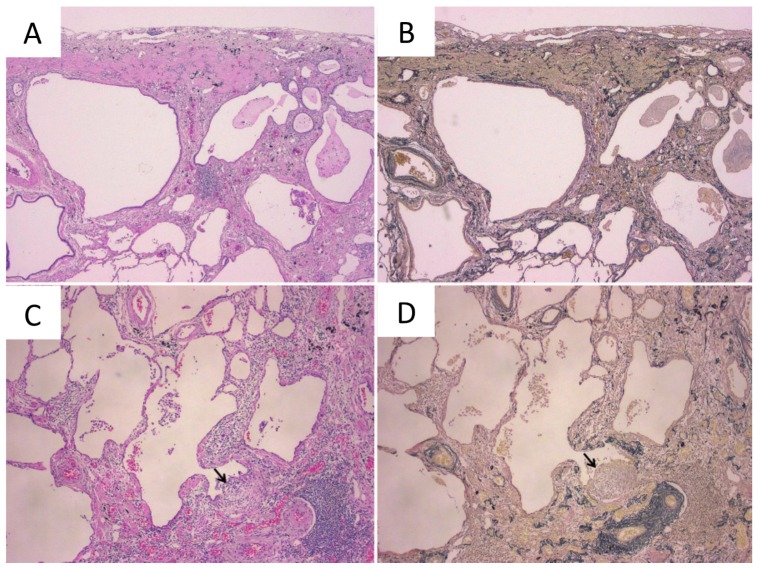
Histopathology of the microscopic usual interstitial pneumonia (UIP) pattern. (**A**,**B**) Microscopic UIP pattern in the background lung. (**C**,**D**) The arrow indicates active fibroblastic foci (**A**,**C**, Hematoxylin and eosin staining; **B**,**D**, Elastica-van Gieson staining). This figure is adapted from Goto T. *Ann Thorac Cardiovasc Surg* (2011) 17, 573–576 [26].

AEIPF of the usual interstitial pneumonia (UIP) pattern is pathologically recognized as a diffuse alveolar damage in UIP [23]. In spite of the absence of any radiological findings of IPF, it does rarely happen that postoperative histology of the background lung reveals a UIP pattern in some patients, and this pattern suggests early-stage IPF and can be defined as microscopic UIP [24,25]. We previously reported a patient who showed AE of microscopic UIP after lung cancer surgery (Figure 1). Even such a microscopic UIP pattern may become exacerbated after chest surgery [26].

The diagnostic criteria for IPF comprise a spatially and temporally heterogeneous appearance of the tissue; alternating zones of normal lung with patchy interstitial widening from a combination of inflammation and fibrosis, active fibroblastic foci, peripheral distribution of fibrotic changes in the lobule, and honeycomb changes (Figure 1) [27]. The diagnosis of IPF necessitates the exclusion of other types of IP, such as interstitial lung diseases associated with a systemic condition, medications, or environmental pathogen exposure [11].

We also reported the presence of IPF to be a risk factor for postoperative mortality, mainly due to the occurrence of AE [18]. In addition, long-term survival turned out to be poor, especially in patients with stage I/II non-small cell lung cancer, with respiratory failure being mainly responsible for death in the late phase [18]. Thus, this microscopic UIP pattern, which cannot be detected by radiological examination, complicates the selection of treatment for lung cancer.

## 4. Risk Factors for Postoperative Acute Exacerbation

A major challenge in the treatment of IP-associated lung cancer is the extremely fatal nature of AE of IP, which is often induced by the cancer treatment itself. For IP-associated lung cancer, radiotherapy is generally contraindicated and the applicability of chemotherapy regimens is also limited. In particular, irinotecan, gemcitabine and amrubicin are, respectively, contraindicated in these patients. Surgery is often selected for resectable tumors in the treatment of such cancer, even though surgery is also a known risk factor for AE. The incidence of postoperative AE of IP varies among patients by their demographics and IP subtypes. Sugiura et al. reported the finding of honeycombing on CT imaging to be a potential risk factor for AE after lung cancer surgery [28]. As earlier reports were generally of a small scale and did not provide a clear definition of postoperative AE, a varying incidence of 0–44.4% and even more widely varying mortality rates of 0–100% are reported. The incidence and mortality of postoperative AEIPF are reviewed in Table 1 [1,2,3,18,29,30,31,32,33,34,35,36,37,38,39,40,41,42,43,44,45,46].

A recent multicenter retrospective cohort study conducted by the Japanese Association for Chest Surgery included 1763 cases, which was much larger and thus expected to provide more reliable findings than previous surveys, reported an incidence of AE in the first 30 postoperative days of 9.3% and mortality rate of 43.9% [43]. This study was intended to clarify the predictive risk factors of AEIPF after lung resection. The study included patients with IP-associated lung cancer diagnosed during a 10-year period from 2000 to 2009 and evaluated the incidence of AE as the primary endpoint and overall survival as the secondary endpoint. The survey consisted of 82 questions regarding patient demographics, as well as factors related to lung cancer, IP and surgery, with information obtained within 1 month of the surgery, including postoperative complications, AE and outcome. The efficacy of AE prophylaxis was unclear. Univariate analysis revealed significant correlations with AE for the following factors: gender, preoperative AE of IP, preoperative history of steroid use, preoperative CRP (C-reactive protein), LDH (lactate dehydrogenase), KL-6 (Krebs von den Lungen-6), %VC (vital capacity), FEV1.0 (forced expiratory volume in 1 s), FEV1.0%, DLCO (carbon monoxide diffusing capacity of the lung), operative time, blood loss, imaging findings of IP, and the surgical procedure used. A subsequent multivariate analysis of these factors identified the following as significant respective risk factors for AE: male gender, preoperative steroid use, KL-6 > 1000 U/mL, %VC < 80%, UIP pattern, history of AE, segmentectomy or a more extensive surgical procedure (reference, wedge resection).

In this study, most of the patients (64.6%) had an onset of AE within 10 days after operation, and postoperative day 4 showed the highest frequency of onset [43]. Compared with the 0.34% postoperative mortality rate for lung cancer reported by the Japanese Association for Thoracic Surgery [47], the mortality rate after surgery for IP-associated lung cancer suggests an extremely high risk in surgery. Moreover, patients with a prior history of AE tend to experience recurrence of AE or continue to suffer from the respiratory dysfunction caused by AE, leading to a poor long-term outcome.

Previously reported risk factors for AE include low vital capacity, low diffusing capacity, high serum CRP, high serum IP markers (e.g., LDH, SP-D, KL-6), longer operative time, thoracotomy rather than thoracoscopy, lobectomy rather than limited surgery, imaging findings of fibrosis, UIP pattern as a subtype of IP, intraoperative fluid balance, and intraoperative inhalation of high-concentration oxygen. However, the definitive risk factors have remained unclear due to the small sample size of previous studies, as mentioned above, and also the variability of the study settings. In the aforementioned large-scale survey conducted by the Japanese Association for Chest Surgery, seven of the aforementioned conventional risk factors were identified as significant independent risk factors for AE [48]: prior history of AE (assigned score 5), UIP pattern (4), anatomical segmentectomy or more extensive surgical procedure (4), male gender (3), preoperative steroid use (3), preoperative serum KL-6 > 1000 U/mL (2) and preoperative %VC < 80 (1). Each of the seven factors was assigned a score (as indicated in parentheses) and the additive scores for each factor were the risk score (RS) for predicting exacerbation. The patients were classified into three groups based on the RS: low risk (RS: 0–10), intermediate risk (RS: 11–14), and high risk (RS: 15–22) groups [48]. This RS has yet to be validated, but is expected to enable a comparison of postoperative AE in patients, matched by background risk factors, and will facilitate evaluation of new preventative and therapeutic approaches as well as identification of appropriate treatment strategies based on risk comparison with other therapies, such as anticancer agents and radiotherapy.

## 5. Strategies for Preventing Acute Exacerbation

### 5.1. Surgery and Selection of the Surgical Procedures

In the aforementioned RS-based exacerbation risk prediction system [48], there is a four-point difference in score between anatomical segmentectomy or more extensive surgical procedure and partial resection. Thus, the selection of partial resection reduces the risk of exacerbation. However, the same survey data also indicate that limited surgery, which increases the risk for cancer recurrence as mentioned earlier, does not provide survival benefit in patients with IP-associated lung cancer [49]. The study also showed that patients with a preoperative %VC of ≤80%, including those in stage IA, had poor prognosis, with a 5-year postoperative survival of 20% [49], suggesting the importance of the preoperative evaluation of the exacerbation risk and selection of an appropriate surgical procedure, as well as careful determination of surgical indication.

Stereotactic radiotherapy (SRT) is increasingly used to treat lung cancer as a substitute for surgery. SRT involves multidirectional irradiation of a small region to enable dose convergence and has demonstrated a treatment outcome comparable to that achieved by surgery in early-stage lung cancer [50]. The impact of interstitial changes on radiation pneumonitis after SRT remains controversial. Some reports show that the presence of asymptomatic IP that is detectable by chest CT is not a significant risk factor for the grade 2–5 radiation pneumonitis caused by SRT [51], whereas other studies suggest the presence of interstitial changes to be an indicator of radiation pneumonitis after SRT [52,53]. Further studies are, therefore, needed to evaluate the efficacy of SRT in treating IP-associated lung cancer.

### 5.2. Intraoperative Management

Hyperextension of the lung caused by positive pressure ventilation with a ventilator and administration of high-concentration oxygen is a known cause of lung injury. Such injury aggravates severely fibrotic lungs with reduced compliance, indicating that hyperoxia, barotrauma, and volutrauma may induce diffuse alveolar damage [54]. During surgery for IP-associated lung cancer, intraoperative management to minimize the intraoperative fraction of inspired oxygen and airway pressure is implemented in many institutions. Nevertheless, the efficacy and safety of these intraoperative management practices remain yet to be evaluated.

### 5.3. Prophylactic Agents

Previous large-scale retrospective studies have included patients treated with steroids, macrolides, ulinastatin, sivelestat sodium, or other agents for the prevention of postoperative AE, although none of these agents has proven to be effective.

As mentioned in the previous section, preoperative steroid use has been identified as a risk factor for exacerbation. Although this may be due to selection bias in favor of severe cases, such as those requiring steroids, multiple previous studies have shown steroid use to be a risk factor for exacerbation [55]. For the treatment of IPF, the most common subtype of IP, the oral anti-fibrotic agent pirfenidone was demonstrated to improve progression-free survival by suppressing progression of IPF in a recent phase III trial [56]. However, the efficacy of pirfenidone in high exacerbation risk settings, such as during the perioperative period, remains to be established. Iwata et al. treated patients with lung cancer associated with IPF with perioperative pirfenidone and noted no serious adverse reactions or occurrence of exacerbation within 30 days of surgery [57,58]. Given that patients treated perioperatively with pirfenidone displayed reduced serum KL-6 levels and reduced intensity of lung injury in resected specimens, it may be possible that the agent exerts its effect by suppressing the perioperative activity of IPF [57,58]. Iwata et al. also conducted a multicenter prospective phase II trial (WJOG6711L: PEOPLE study) and reported favorable results, where only two of 39 eligible patients (5.1%) and one of 36 patients who completed the protocol (2.8%) experienced exacerbation [59]. However, this study was conducted using a single-arm design with a small sample size [59] and the results need to be verified in a larger-scale phase III trial. Nintedanib is another agent with IPF treatment efficacy reported in a phase III trial, but its label recommends discontinuation of its use in the perioperative period due to its potential to delay wound healing. Therefore, when using nintedanib for the prevention of postoperative AE, careful review of the information on adverse reactions must be performed to confirm the safety of the perioperative use of the agent.

## 6. Postoperative Outcome and Prognostic Factors in Interstitial Pneumonia-Associated Lung Cancer

**Figure 2:**
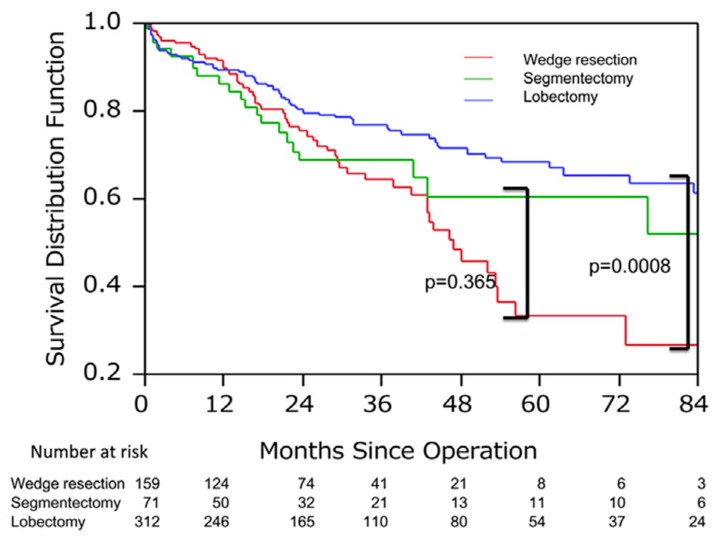
The survival associated with the surgical procedure in patients with stage IA interstitial pneumonia (IP)-associated lung cancer. 5-year survival of the wedge resection group (29.2%) was significantly worse than that of segmentectomy (60.0%) or lobectomy group (68.6%). This figure is adapted from Sato T. J Thorac Cardiovasc Surg (2015) 149, 64–70 [49].

Few investigators have reported on the long-term outcome after lung resection for non-small cell lung cancer patients with IPF. A large-scale survey conducted by the Japanese Association for Chest Surgery showed that the 5-year postoperative survival of IP-associated lung cancer was as low as 40% in the entire population and 59% in those with stage-IA disease [49], which are much worse survival rates than those reported in a survey conducted by the Japanese Joint Committee for Lung Cancer Registration (86.8% for stage IA). Multivariate analysis identified wedge resection, %VC < 80% and tumors located in the inferior lobe as poor prognostic factors. When analysis was limited to stage-IA patients, the survival curves for those treated with wedge resection and those treated with lung lobectomy crossed at approximately 1 year after surgery, with a 5-year survival of 29.2% with wedge resection and 68.6% with lobectomy (*P =* 0.0008, Figure 2). These results demonstrate that wedge resection is associated with a lower incidence of AE and better short-term outcome, but with a higher rate of cancer recurrence and worse long-term outcome compared to lobectomy. An appropriate strategy must be adopted, such as selecting wedge resection for patients at high risk of postoperative AE and anatomical curative resection for lower-risk patients. It should be noted that adjuvant chemotherapy is not recommended, due to its limited efficacy and the potential risk for inducing AEIPF. Thus, these patients are generally treated with surgery alone.

Watanabe et al. reported that the 5-year survival rates were 61.6% in pathological stage IA lung cancer patients with IPF and 83.0% in those without IPF. In addition, 5-year recurrence-free survival was 56.0% and 83.1%, respectively [3]. Likewise, Saito et al. reported that 5-year survival rates for pathologic stage I lung cancer were significantly different between patients with IPF (54.2%) and those without IPF (88.3%). [1]. These results and other reported data pertaining to the long-term outcome are reviewed in Table 2 [1,2,3,17,31,60,61]. Lung cancer in the presence of IP may be associated with smoking as a background factor and be of high biological malignancy. The lungs of patients with IP are at high risk for cancer development and also appear to be at increased risk for recurrent multiple cancers after the initial resection [4,62,63]. IP itself is generally associated with progression of IP and poor prognosis; it is noteworthy that death from AE and subsequent respiratory failure also affect the prognosis of patients with IP-associated lung cancer [31,49]. IP may also limit treatment options for recurrent lung cancer, precluding the use of aggressive anticancer treatment and/or radiotherapy [18]. These complex factors are likely to contribute to the poorer prognosis of lung cancer patients with IP than those without IP.

## 7. Conclusions

For the surgical treatment of IP-associated lung cancer, surgical indication and surgical procedures should be carefully determined based on the available evidence. Recent studies have offered additional data regarding the outcome, incidence of postoperative AE and risk factors associated with surgery for IP-associated lung cancer. Promising drugs are available for the prevention of postoperative AE, but require further validation. Developing a safe and effective treatment for IP-associated lung cancer is an urgent task. A new treatment strategy based on the biological or genomic characteristics of IP should be established through novel translational research and large-scale clinical trials.

## Figures and Tables

**Table 1:** Reports on postoperative acute exacerbation of idiopathic pulmonary fibrosis in non-small cell lung cancer patients with idiopathic pulmonary fibrosis.

Author	Ref.	Published Year	No of Pts with IPF	No of Pts with AEIPF	Incidence of AEIPF (%)	Death of AEIPF	Mortality of Pts with AEIPF (%)
Fujimoto	[32]	2003	21	0	0.0	0	0.0
Kumar	[35]	2003	24	5	20.8	4	80.0
Chiyo	[31]	2003	36	9	25.0	3	33.3
Koizumi	[34]	2004	47	7	14.9	6	85.7
Okamoto	[39]	2004	20	4	20.0	3	75.0
Kushibe	[36]	2007	33	4	12.1	4	100.0
Watanabe	[3]	2008	54	4	7.4	4	100.0
Chida	[30]	2008	91	11	12.1	7	63.6
Minegishi	[37]	2009	35	8	8.6	3	37.5
Shintani	[44]	2010	40	6	15.0	5	83.3
Yano	[46]	2011	7	1	14.3	0	0.0
Suzuki	[45]	2011	28	9	32.0	0	0.0
Saito	[1]	2011	28	3	11.7	0	0.0
Park	[42]	2011	100	28	28.0	13	46.4
Chida	[29]	2012	52	6	11.5	3	50.0
Mizuno	[38]	2012	52	7	13.5	6	85.7
Voltolini	[2]	2013	37	5	13.5	3	60.0
Sato	[43]	2014	1763	164	9.3	72	43.9
Goto	[18]	2014	65	4	6.2	4	100.0
Omori	[40]	2015	103	5	4.9	3	60.0
Joo	[33]	2016	80	6	7.5	-	-
Otsuka	[41]	2016	9	4	44.4	3	75.0

Ref: reference, Pts: patients, IPF: idiopathic pulmonary fibrosis, AEIPF: acute exacerbation of IPF.

**Table 2:** Reports on the long-term outcome of non-small cell lung cancer patients with idiopathic pulmonary fibrosis after lung resection.

Author	Ref.	Year	Pts with IPF	Pts without IPF	5-Year Survival	
					IPF (+)	IPF (−)
Kawasaki	[60]	2002	53	658	53.0	67.5
Chiyo	[31]	2003	36	895	35.6	71.0
Watanabe	[3]	2008	56	802	61.6	83.0
Saito	[1]	2011	28	322	54.2	88.3
Voltolini	[2]	2013	37	738	52.0	65.0
Sekine	[61]	2014	41	339	36.9	66.1
Lee	[17]	2014	33	66	73.0	38.0

Ref: reference, Pts: Patients, IPF: idiopathic pulmonary fibrosis.
